# The Treatment of Carcinoma of the Cervix

**Published:** 1903-05

**Authors:** Virgil O. Hardon

**Affiliations:** Atlanta, Ga.


### THE TREATMENT OF CARCINOMA OF THE CERVIX.

VIRGIL O. HARDON, M.D.,

ATLANTA, GA.

The purpose of this paper is not to offer any new treatment of cancer of the cervix, but simply to emphasize certain points which are already well-established but which appear to have escaped the attention of many practitioners of medicine.

The wonderful progress which has been made in the science of medicine during the last half century has increased the average duration of life by about ten years. This has been effected by lessening the mortality in many of the diseases to which flesh is heir, and by diminishing the frequency of many diseases by sanitary and other measures.

But unfortunately cancer of the cervix cannot be included in either of these categories. The statistics gathered by Baldy have shown that under the most approved method of treatment a permanent cure cannot be hoped for in more than five per cent. of cases. Statistics also appear to show that there has been a marked increase in the frequency of malignant growths in the last fifty years. Hence, at first glance the treatment of cancer of the cervix would appear to present a most discouraging aspect. But when it is remembered that it is but a short time since every case of this disease inevitably terminated in death, a saving of five cent. of cases must be recognized as a distinct gain. I shall also presently show that in spite of the greater frequency at the present day, something has been accomplished in the way of prevention, so that the increase has been less decided than it would have been if preventive treatment had not been adopted.

It may seem a bold assertion to say that cancer of the cervix is a preventable disease. Yet in the light of our present knowledge the statement is unquestionably true. A discussion of the prevention of the disease necessarily involves the study of its causation, and therein lies the whole gist of the matter. It is now a quarter of a century since Emmet first advanced the theory that cancer of the cervix is due to laceration in labor. He said "One fact we may accept beyond question, and that is, the occurrence of this form of malignant disease in a woman who has never been impregnated must be exceedingly rare." In his recent work on Cancer of the Uterus, Cullen reiterates the same idea, and says that in fifty of his cases of carcinoma of the cervix, in which accurate data were available, forty-nine had had children. No careful and competent observer from Emmet to Cullen has failed to corroborate these views. That laceration of the cervix is the determining factor is shown by the statement of Emmet, that he has never failed to demonstrate the existence of such laceration in cases where the disease had not yet involved the whole cervix. It is also shown by the fact, noted by Emmet, by Kelly and by others, that in most of the rare cases in which carcinoma has occurred in the cervices of women who had not conceived, there was a history of instrumental dilatation of the cervix to account for it. Boldt, in one of his cases, was able to prove beyond question that the original point of attack was in the depths of a laceration of the cervix. The long-continued irritation of the cervical tissues by Nature's efforts at repair is the cause of the perversion of nutrition which results in carcinomatous degeneration.

If these statements be true, as they have been proven to be by a quarter of a century of observation, it is obvious that the prophylaxis of cancer of the cervix consists in the timely repair of cervical lacerations. The proposition may be laid down without fear of successful contradiction, that if every laceration of the cervix were subjected to operation before the tissues involved had taken on malignant changes, cancer of the cervix would soon become as notable for its rarity as it now is for its frequency. The importance of this conclusion cannot be overestimated. If thousands of women die every year from a disease which can be prevented by a well-known treatment based upon well-established principles, that fact is a serious reflection upon the profession of medicine. The great frequency of cancer of the cervix would seem to argue a culpable ignorance or a still more culpable negligence on the part of the profession in general. The diagnosis of a laceration of the cervix does not require the skill of an expert. The tyro in medicine can recognize it. If every physician would examine every woman a month after he has attended her in labor, and, if a laceration is found, would warn her of the danger of allowing it to go unrepaired, cancer of the cervix would become almost a thing of the past.

When cancer of the cervex is already present the question of how it shall be treated constitutes a grave problem which requires separate settlement for each individual case. The cases which present themselves may be divided, with reference to treatment, into three classes:

1. Operable cases which offer a possibility of permanent cure.
2. Operable cases which offer no hope of permanent cure.
3. Inoperable cases.

Unfortunately the number of cases in the first class, the operable cases which offer a possibility of permanent cure, is exceedingly limited. This fact is due to the obscure and insidious character of the first symptoms of the disease. But not entirely to this cause. Here again the general practitioner is often at fault. The first symptom of cancer of the cervix is, in the majority of cases, menorrhagia, or metrorrhagia. The patient often ignores this symptom in the absence of pain or other indication of disease. If she reports it to her physician, he will often ascribe it to the change of life, or otherwise treat it as a trivial matter. When, later on, the advent of pain and foul discharge indicates to the patient the existence of serious disturbance in her pelvis, she again seeks medical advice, and her physician then recognizes the serious nature of the case. He sends her to a specialist for operation, only to be told that it is too late. Thus a valuable life is sacrificed through the ignorance or the negligence of the physician. This is not a fancy picture, nor is it an infrequent one. If every physician would investigate by vaginal examination every case of ab-abnormal menstruation, especially in women over forty years of age, and when in the slightest doubt send the patient to one whose experience will enable him to resolve the doubt, the number of operable cases which offer a hope of permanent cure would be materially increased.

What cases belong in this first class? There is no rule by which a strict line of demarcation can be drawn. In a general way it may be said that those cases in which the disease has not extended beyond the cervix offer the best prospect for permanent cure. Yet even in the majority of these cases recurrence will follow operation. The lymphatic glands of the pelvis become involved very early in the disease. Such involvement precludes all possibility of permanent cure. Any one who has studied the location of the deeper glands of the pelvis need not be told of the anatomical impossibility of their entire removal. In the majority of cases which seem most favorable for operation recurrence will take place. It is also true that a few of the cases which seem less favorable for operation will offer an agreeable surprise by remaining free from recurrence. I would incidentally remark, in this connection, that every case of permanent cure is open to the suspicion of error of diagnosis, unless the presence of carcinoma has been proven by expert microscopical examination.

In the second class, operable cases which offer no hope of permanent cure, are included those in which breaking down of cervical tissue has taken place, or the induration has extended beyond the cervix into the vaginal vault, but in which the womb still remains movable. Mobility of the womb should, in my opinion, be regarded as the decisive test between operable and inoperable cases. When the cancer has reached the stage of invasion of the vaginal wall, or of ulceration, the pelvic lymphatic glands will inevitably have become involved, and a permanent cure is impossible. What benefit, if any, may be expected from operation in such cases? It may be confidently expected that life will be prolonged, and that even though recurrence takes place, the symptoms will be much less distressing than if no operation be undertaken. After removal of the womb by vaginal hysterectomy, the period of immunity from the disease may extend over several months, and even years, a much longer duration of life than could possibly be expected if the disease had been allowed to pursue its natural course. Such prolongation of life is a desideratum, at least from the patient's point of view, and constitutes a legitimate object of treatment. Not only does removal of the womb in hopeless cases prolong life, but when recurrence finally takes place it is accompanied by less distressing symptoms than are present in those cases in which the womb has been allowed to remain. There is much less tendency to hemorrhage. One of the physiological functions of the womb is periodical bleeding. Any pathological derangement of structure tends to corresponding derangement of function, and therefore the diseased womb bleeds more freely and with less provocation than any other of the pelvic tissues. There is also less of the stinking discharge which is so annoying to the patient and her friends. This offensive odor is due in part to the altered secretions of the muciparous glands of the uterus, and in part to the decomposition of broken-down tissue. That it is not entirely due to the latter cause is shown by the fact that it is often present before breaking down of tissue occurs. If the womb be removed recurrence is accompanied by a discharge which is less in quantity and less offensive in odor, a very distinct gain to a patient of delicate sensibilities. Clinical experience has also shown that recurrent cancer after hysterectomy is accompanied by less pain than the original disease. For these reasons vaginal hysterectomy is, in my judgment, indicated whenever the womb is still movable, and the operation is therefore practicable.

The cases reported by Byrne, of Brooklyn, seem to show that the results, as regards permanent cure, obtained by high amputation of the cervix with the cautery knife, compare favorably with the results of total hysterectomy. But since a permanent cure can be hoped for in only five per cent. of cases, under either plan of treatment, it is the part of wisdom to select the operation which will be most beneficial to the ninety-five per cent. of cases in which recurrence is to take place. For this reason total vaginal hysterectomy has been almost universally adopted as the operation of choice. I shall not describe the technique of this operation, but will only say that my experience has led me to prefer the clamps to the ligature as giving greater facility of operation and less danger of hemorrhage. The mortality from the operation is very small. In my own experience I have been so fortunate as to have one hundred per cent. of recoveries up to the present time. But the operation seems to have a small inherent mortality which will probably show itself in my practice sooner or later.

In the treatment of inoperable cases much can be accomplished to prolong life and lessen suffering. It is absolutely inexcusable to leave such cases untreated with the statement that nothing can be done. The great majority of women who die from cancer of the cervix die from the chronic septicemia induced by the absorption of septic matter from the diseased tissues and from the decomposing débris of ulcerated surfaces. To this cause are chiefly due the cachexia, the rise of temperature, the loss of strength and appetite, and the emaciation which characterize the later stages of the disease. The prevention of septicemia by appropriate treatment will enable the patient to maintain a comparatively good general condition almost up to the occurrence of the inevitable death. By such treatment life may be prolonged for months, and in some cases even for years. My plan of treatment consists of frequent removal of all broken-down tissue with the sharp curette and the daily application of antiseptic solutions to the ulcerated surface of the cervix. I formerly used tampons of absorbent cotton, saturated with a solution of chloral hydrate, and then rolled in iodoform. But I found that the odor of iodoform interfered with the appetite of the patient, and I now use boric acid. The patient can be taught to use these tampons herself. Under this treatment I have seen women who were bed-ridden and apparently almost moribund, gain strength, appetite and flesh, rise from their beds, and live for months in comparative comfort. Pain can be relieved by morphine, which should be given without hesitation in sufficient doses to accomplish its purpose. Hemorrhage can be neither foreseen nor prevented. When it occurs it may usually be controlled by tamponade of the vagina. I have found that severe hemorrhages are much less liable to occur in cases which are subjected to frequent curetting. In this class of cases death is, of course, inevitable. But by adopting the methods which I have described much may be accomplished in postponing the fatal issue and in smoothing the patient's pathway to the grave.

The points which I desire particularly to emphasize are—

1. The importance of early repair of all lacerations of the cervix as a preventive measure.

2. The importance of early recognition and treatment of the disease in order to increase the chance of a permanent cure by operation.

3. The advisability of hysterectomy in operable cases which offer no prospect of a permanent cure.

4. The value of palliative treatment in inoperable cases.

